# Advance care planning for patients with advanced multiple long-term conditions: a qualitative thematic analysis of health and social care professionals’ perspectives

**DOI:** 10.1186/s12904-026-02173-2

**Published:** 2026-06-04

**Authors:** Georgina Simpson, Felicity Dewhurst, Philip Mordue, Charlotte Stenson, Mark Adley, Katie Brittain, Barbara Hanratty

**Affiliations:** Population Health Sciences Institute, Faculty of Medical Sciences, Campus for Ageing and Vitality, Newcastle University, Newcastle-upon-Tyne, NE4 5PL UK

**Keywords:** Advance care planning, Multimorbidity, Multiple long-term conditions, Continuity, Equity

## Abstract

**Background:**

Advance care planning helps people plan for and communicate preferences about future care, including when decision-making capacity is lost. For people living with advanced multiple long-term conditions, advance care planning is challenging because decline may be unrecognised, responsibility is diffused across condition-specific services, and care is fragmented.

**Aim:**

To explore health and social care professionals’ perspectives on challenges and best practices in advance care planning for people with advanced multiple long-term conditions, and to generate recommendations for improving practice.

**Methods:**

Reflexive thematic analysis of advance care planning-related interview data from a wider study examining palliative care needs and end-of-life challenges for people with multiple long-term conditions. Twenty-seven semi-structured interviews were undertaken with health and social care professionals, commissioners, and service managers in four regions of England.

**Results:**

Three themes were generated: (1) Making multiple long-term conditions decline visible – cumulative illness burden and social adversity contributed to late recognition of deterioration; (2) System and organisational barriers – resource constraints, fragmented care, poor information sharing, and limited training created unclear ownership of advance care planning, with particular risks for people experiencing inequity; (3) Delivering advance care planning – effective advance care planning was introduced early, revisited iteratively, grounded in trusting relationships, and included families and care networks.

**Conclusions:**

For advanced multiple long-term conditions, advance care planning is undermined by prognostic uncertainty, care fragmentation, and inequities. Embedding prompted, iterative advance care planning into routine care, using markers such as functional decline and escalating support needs, may normalise conversations and support earlier documentation of preferences. This analysis extends needs-focused work by specifying practical mechanisms to initiate, share ownership of, and sustain advance care planning across fragmented services.

**Supplementary Information:**

The online version contains supplementary material available at 10.1186/s12904-026-02173-2.

## Introduction

Multiple long-term conditions (MLTCs), defined as the coexistence of two or more chronic illnesses, are a growing international health challenge, driven by population ageing and a rising prevalence of chronic disease [1–3]. People living with MLTC commonly experience frailty, functional decline, polypharmacy, and frequent acute care use, which often become more problematic as conditions progress [3]. Recent work has introduced the term *advanced multiple long-term conditions*, conceptualising it as deteriorating health and the point when individuals may be approaching end of life and/or could benefit from a palliative care approach alongside ongoing disease management [4]. In this context, trajectories in MLTC are often uncertain, with alternating periods of relative stability and episodes of sudden deterioration, creating distinctive challenges for timely care planning and decision-making [4, 5].

Health and care systems have largely evolved around single-disease specialties and condition-specific pathways, with care organised through episodic contacts, referrals, and transitions between settings [6, 7]. Care often begins in generalist settings (such as primary care) before people are referred into specialist services structured around single conditions; later, when holistic, integrated support is needed, systems can struggle to bring fragmented pathways back together [6, 7]. For people living with MLTC, this can create ambiguous responsibility, such that no single clinician or team consistently holds longitudinal oversight for initiating and revisiting forward-looking discussions. In practice, changes in health status may be missed because cues are distributed across settings and accumulate over time, including escalating frailty, recurrent unplanned admissions or “near-miss” crises, increasing symptom burden, accumulating polypharmacy and treatment burden, and growing caregiver strain. When these cues are not routinely identified, shared, and acted upon across services, planning is more likely to be reactive than anticipatory [8, 9].

Advance care planning (ACP) provides a framework for addressing these challenges by enabling individuals to define goals and preferences for future treatment and care, discuss these with family and healthcare professionals, and record and review preferences if appropriate. ACP is conceptualised internationally as an ongoing, iterative process involving individuals, those important to them, and healthcare professionals, rather than a single event or document [10–12]. In conditions characterised by uncertain and fluctuating illness trajectories, such as MLTC, ACP can be initiated in response to emerging patterns of decline and evolving need, rather than relying on prognostic timing [5].

Evidence syntheses indicate ACP interventions can improve communication and documentation and are associated with greater alignment between delivered care and patients’ stated priorities, although effects vary by intervention type, outcome domain, and setting [13, 14]. Within the literature there is also criticism of ACP on the grounds that it can become reduced to administration or documentation processes in which system needs are prioritised over the needs of patients, particularly if ACP is implemented without meaningful engagement with patients’ values, cultures, and lived experiences [15–17]. In this study, we conceptualise ACP as a relational, values-based and iterative process, which may include documentation such as treatment escalation plans. This distinction is particularly important for people living with MLTC, where uncertainty around trajectories challenges traditional, prognosis-driven approaches to care planning.

MLTC adds a distinct layer of complexity because deterioration is distributed across interacting conditions and care is often delivered through multiple specialties and services [18]. This can diffuse responsibility for ACP, generate competing treatment priorities, and make it harder to recognise when to shift towards anticipatory, goal-focused care. These issues also arise when cancer co-exists with other long-term conditions, where additional conditions can increase complexity and healthcare use beyond a single-disease pathway [19]. Similar challenges are described in dementia, where cognitive decline increases reliance on surrogate decision-making and can heighten the importance of earlier planning [20, 21].

For people living with MLTC, ACP may therefore be especially pertinent because acute exacerbations can trigger unplanned admissions and treatment escalation before goals and preferences have been discussed or documented [22], and because documented goals can provide a shared reference point during transitions and handovers across teams and settings [11, 14]. Yet despite these potential benefits, ACP remains inconsistently delivered for people living with MLTC [23]. Prognostic uncertainty is often treated as a reason to defer conversations rather than as a prompt to start them, and fragmented pathways and competing priorities can leave responsibility for ACP unclear [20, 24, 25].

While research has explored ACP in conditions predominated by single diseases, less is known about how ACP is understood and enacted for people living with MLTC, particularly across sectors. Few studies have examined the perspectives of health and social care professionals spanning different settings on how ACP could be improved for this patient group. This paper reports a reflexive thematic analysis of ACP-related interview material from a wider study examining palliative care needs and end-of-life challenges for people living with MLTC, with the aim of identifying challenges, best practices, and actionable recommendations to strengthen ACP delivery.

### Aim

This paper explores health and social care professionals’ perspectives on the challenges and best practices of ACP for people with advanced MLTC, generating recommendations for improving delivery.

## Methods

### Design

We undertook a qualitative interview study using a multiple case study design and reflexive thematic analysis [26–28], prioritising insights with direct relevance to practice and policy for people with advanced multiple long-term conditions. The analytic stance was interpretivist, treating accounts as situated and shaped by participants’ roles and service context [26–28]. Reporting followed the COREQ checklist [29] (Supplementary Material 1).

### Data source

Data comprised semi-structured interviews collected within a broader study examining palliative care needs and end-of-life challenges for people living with MLTC. This paper reports an ACP-focused analysis of that dataset. The interview topic guide is attached as Supplementary Material 2.

### Settings, sampling and participants

Four English regions (Blackpool, Kent, Northumberland and Yorkshire) were selected as case study settings to capture variation in geography, socioeconomic deprivation and ethnic diversity. The original study used purposive sampling to ensure diversity across sectors and professional roles. Potential participants were identified through professional and service networks across the four study regions, including clinical, commissioning and voluntary sector contacts. Participants were approached by email with an information sheet. No invited participants declined or withdrew from the study: all 27 interviews with health and social care professionals from these settings were included in the present analysis.

### Data collection

Virtual interviews were conducted by CS (female; MBBS, MRCP(UK), MSc; NIHR Palliative Care Academic Clinical Fellow) and PM (male; NIHR ARC NENC Knowledge Mobilisation Fellow/Integrated Care Board Research Manager; PhD candidate in Public Health), both trained and experienced in qualitative interviewing. Participants were informed of the interviewers’ roles and the study purpose. No relationship was established prior to recruitment, aside from routine clinical contact where relevant. Interviewers’ professional interest in improving ACP for people with MLTC was acknowledged and considered through reflexive field notes.

A semi-structured topic guide was developed by the research team, informed by relevant literature and clinical and service experience, and refined iteratively after early interviews. Interviews lasted between 30 and 120 min, reflecting the varying amount of participant availability. Interviews were audio-recorded with consent, professionally transcribed verbatim and pseudonymised. The semi-structured topic guide was developed by the research team, informed by existing literature and clinical experience, and was refined iteratively following early interviews to enhance focus and relevance. This iterative approach is consistent with reflexive thematic analysis, in which data collection evolves in response to the researcher’s developing understanding, allowing early interviews to function in place of formal pilot testing [26, 30].

Interviews explored how deterioration was recognised, how planning conversations were initiated, how ACP was coordinated across services, and perceived barriers and enablers. Reflexive field notes were completed after interviews to document contextual observations and emerging analytic insights. No repeat interviews were conducted. Interviews were conducted remotely using Microsoft Teams between July 2024 and February 2025, with participants typically joining from home or their workplace. No non-participants were present. Transcripts were not returned to participants. Recruitment ceased when a point of saturation was reached, in terms of no new concepts or perspectives being identified relating to the wider study’s topics of palliative care needs, service provision, and end-of-life challenges for people with MLTC [27].

### Analysis and reflexivity

We conducted reflexive thematic analysis following Braun and Clarke’s six phases [26, 28]. Transcripts were read for familiarisation, coded inductively and then refined iteratively with reference to the study aims; NVivo 15 supported data management [31]. Analysis was led by GS (clinician-researcher with professional experience in community nursing and palliative care) as part of her Master of Public Health dissertation, including qualitative methods training. GS undertook primary coding and developed candidate themes, with BH and FD providing weekly supervision and actively reviewing coding decisions and theme development. BH and FD each reviewed a subset of transcripts and associated coding and discussed theme boundaries and interpretations with GS to support critical challenge and alternative readings of the data. An audit trail of analytic memos and reflexive field notes supported transparency. Themes were derived from the data rather than specified in advance.

Analysis primarily identified shared patterns across sites; although settings were selected to capture contextual diversity, no consistent between-site differences were observed in how ACP was described. Consistent with reflexive thematic analysis, coding reliability was not calculated and participant checking of findings was not undertaken.

### Trustworthiness

We drew on established principles of qualitative trustworthiness [32]. Credibility was supported through iterative coding and critical discussion of developing interpretations. Transferability was strengthened through purposive diversity across settings, roles and sectors and contextual description of the case study settings. Dependability and confirmability were supported through systematic data management in NVivo, an audit trail of analytic memos and reflexive documentation, with regular discussion within the research team. Quotations are used to illustrate themes and are labelled with participant identifiers (site and role). Interpretations were checked against coded extracts and transcripts to ensure coherence between data and findings. Major themes are presented clearly in the Results with supporting quotations. Minor themes and deviant cases are not presented separately; the Results focus on the dominant patterns across interviews.

### Theoretical framework

The study was conducted within a theoretical framework of person-centred care, which emphasises understanding individuals’ values, priorities and lived experience, rather than focusing solely on disease-specific outcomes [33]. In the context of MLTC, this is closely linked to concepts of treatment burden, where the cumulative work of managing multiple conditions can outweigh perceived benefits [34]. In the context of ACP, continuity of care plays a key role in person-centred care, supporting the development of shared understanding and trust over time [35].

### Data availability

Interview transcripts are not publicly available due to confidentiality agreements. Anonymised extracts are presented in the Results.

## Results

### Participant characteristics

Twenty-seven interviews with health and social care professionals were included in the present analysis. We defined health and social care professionals as individuals involved in commissioning, managing, planning, or delivering services within statutory or voluntary, community and social enterprise (VCSE) organisations. Table 1 summarises participant characteristics by role category and site.

**Table 1:** Participant characteristics (*N* = 27)

Role category	Roles included	Number of participants *
General practitioners (GP)	GP leads for palliative care, out-of-hours GPs, clinical advisers	5
Medical specialists	Geriatrician, hospital palliative care consultant, community palliative medicine consultants	4
Nursing staff	Community nurses, specialist palliative care nurses, community matrons	7
Allied health professionals (AHP)^a^	Occupational therapist, AHP lead, advanced clinical practitioner^b^ in acute response team^c^, social worker and lead for continuing healthcare funding	2
Paramedics	Advanced paramedic, advanced clinical practitioner coordinator	2
Hospice leaders^d^, Chief Executive Officers (CEO)	Hospice directors, CEOs, clinical leads	3
Commissioners and service managers	Integrated care board commissioners^e^, service managers, continuing care leads	3
Voluntary/third sector leaders	Older person service lead service lead, public health end-of-life community engagement lead	2
Total		27

^a^Regulated, degree-level clinical practitioners - separate from doctors, nurses, and dentists - who provide specialised, patient-centred care

^b^Registered healthcare professionals working at a high level of autonomy

^c^Community-based multidisciplinary team providing healthcare and rehabilitation at home

^d^Specialised palliative care services with inpatient units and community services

^e^NHS organisation responsible for planning and commissioning local services

*Several participants held multiple roles of interest (e.g. clinician with commissioning responsibilities)

### Qualitative interviews

**Fig. 1:**
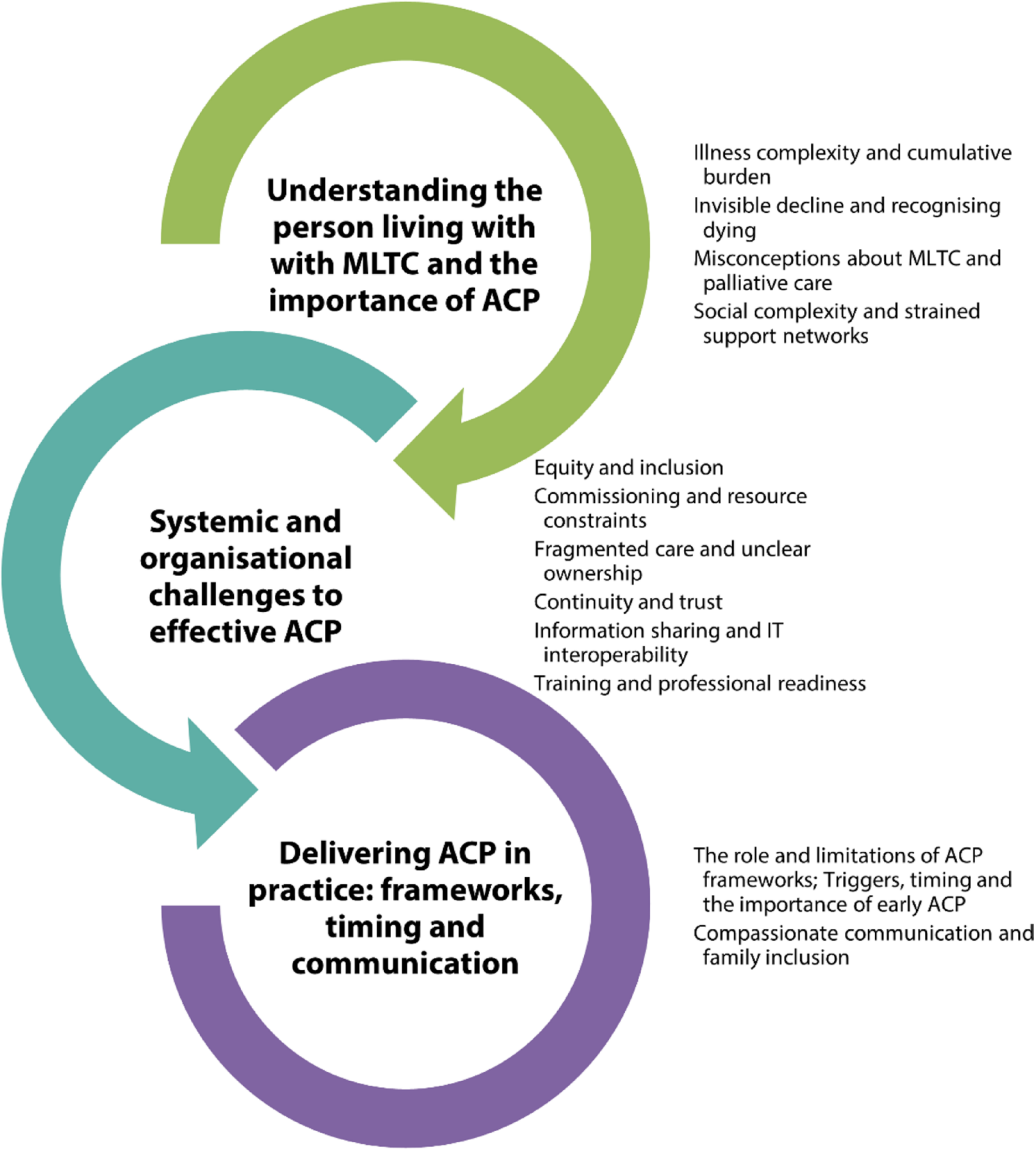
Themes and subthemes

Analysis generated three overarching but inter-related themes with related subthemes (Fig. 1).

These describe how professionals understood ACP for people living with MLTC, the challenges encountered, and examples of effective practice. Accounts were broadly consistent across sites.

### Theme 1: Understanding the person living with MLTC and the importance of ACP

#### Illness complexity and cumulative burden

Participants highlighted the cumulative impact of living with multiple conditions, where trajectories were non-linear and unpredictable. Rather than one dominant illness, decline was often dispersed across bodily systems and manifested as frailty and functional decline.

*“Their [individuals with MLTC] needs are not met in current services*,* so that’s a problem in itself. And then it’s also that it is not even double trouble with two diagnoses. It’s with more than two diagnoses they start to compete with each other*,* and it makes it more complicated in terms of management of the conditions.” Participant 1*,* Community Palliative Medicine Consultant*.

This complexity made it difficult for professionals to frame prognosis or decide when ACP should be initiated. Participants described the need to rely less on predicting time to death and more on recognising changes in function, increasing treatment burden, and repeated crises (for example, hospital admissions) as signals that ACP should start and be revisited over time.

*“[In hospital] we’ll have opportunities to do advanced care planning*,* but in reality*,* I do very little there because they’re in a phase of life where you’re not quite sure where they’re going to be. And so having that conversation is difficult.” Participant 3*,* GP Lead for Palliative and End-of-Life Care*.

Participants also described the lack of clear “markers” in MLTC compared with some single-disease pathways, making timing feel uncertain and “nebulous”.

*“[With cancer] there can be quite clear markers… it’s almost quite binary in that way. Whereas I think with multiple long-term conditions*,* it’s a lot more nebulous.” Participant 4*,* Head of Service for Palliative Care*.

Examples were given where formal planning only occurred after an acute deterioration, with the quote below referring to a Do Not Attempt Cardiopulmonary Resuscitation form (DNACPR) advising that cardio-pulmonary resuscitation should not be attempted if a person’s heart or breathing stops.

*“[The patient] become really septic and had been readmitted*,* and it was that admission that the DNACPR was put in place.” Participant 3*,* GP Lead for Palliative and End-of-Life Care*.

ACP in this context needed to address not only disease management but the overall burden of illness and treatment, including daily functioning, quality of life, and the person’s priorities.

#### Invisible decline and recognising dying

Professionals described how decline in MLTC was often missed. This was attributed to uncertainty in recognising cumulative deterioration and a tendency to focus on managing acute episodes rather than acknowledging gradual decline.

*“[In MLTC] you usually recognise when someone’s very towards the end of life*,* but it’s the bit in between that we miss.” Participant 3*,* GP Lead for Palliative and End-of-Life Care*.

This contributed to late ACP, often initiated only during crises. Participants argued that functional losses and increased care needs should be used as earlier prompts, shifting the focus from predicting death to recognising lived decline.

Participants also emphasised the importance of initiating ACP outside acute episodes, when people could engage meaningfully.

*“So*,* it’s about trying to have those discussions outside of a crisis*,* because it’s no good going out to somebody with an exacerbation of COPD who’s tripoding […] really struggling*,* and going*,* ‘Right*,* have you thought about…’” Participant 18*,* Matron*,* Acute Response Team*.

#### Misconceptions about MLTC and palliative care

Participants described misconceptions that limited timely ACP, including assumptions that palliative care and its components are cancer-specific and uncertainty about when palliative approaches are appropriate for people living with MLTC. These misconceptions were described as operating at multiple levels (professional, service and societal), contributing to delayed recognition and referral.

*“Patients*,* when you say the word ‘palliative’ […] do tend to still think cancer […] they don’t necessarily see themselves as having a palliative condition.” Participant 15*,* Hospice Clinical Director*.

Some participants suggested that scepticism could also arise from repeated prior “false alarms” over years of chronic illness, making it harder to engage people in planning.

*“I’ve had this condition for 20 years*,* and they’ve told me I’m going to die loads of times before*,* and that’s never happened […] ‘But we really mean it this time.’ Yeah*,* they said that last time as well.” Participant 4*,* Head of Service for Palliative Care*.

Participants also reported that these challenges could be amplified when people living with MLTC were younger, with ACP described as unexpected and emotionally difficult, and therefore more likely to be deferred.

*“Talking to people with multimorbidity who are young is one of the biggest ones that we need to crack […] talking to younger people about their wishes is really*,* really challenging*,* and I just don’t think we’re doing it at all.” Participant 14*,* GP Clinical Advisor for Palliative and End-of-Life*.

#### Social complexity and strained support networks

ACP in MLTC was shaped by social circumstances, including long-term caregiving, socioeconomic hardship, and support networks that became strained over time. Participants described how prolonged caring roles could lead to exhaustion, limiting families’ capacity to engage in future planning.

*“Carers are often burned out by the time we get to this point because they’ve been caring for such a long time.” Participant 6*,* Occupational Therapist*.

Immediate pressures, such as finances and daily survival, often overshadowed future planning.

*“In some parts […] everybody’s too worried about how they’ll feed their kids next week to give two shits (about planning for the future).” Participant 12*,* Hospital Consultant*.

These accounts underscored the importance of ACP as an ongoing, relational process tailored to social and family context.

### Theme 2: Systemic and organisational challenges to effective ACP

#### Equity and inclusion

Professionals described inequities in access to ACP, with socioeconomically deprived and ethnically diverse communities less likely to be given opportunities for future planning. Language barriers, mistrust, and limited confidence in culturally sensitive practice were described as restricting engagement. Participants emphasised that inequities were shaped less by patient reluctance than by societal norms around talking about dying and a lack of trust in health and social care professionals.

*“The barriers for the Muslim population in trust*,* like*,* they’re only just starting to get some trust back after the COVID situation. So*,* they might not even access primary care or trust the care that they’re receiving as a start.” Participant 14*,* GP Clinical Advisor for Palliative and End-of-Life*.

Several participants also stressed that these inequities reflected systemic failings rather than patient unwillingness. In more deprived areas, professionals observed that people were less likely to be supported in understanding or accessing ACP, not due to their lack of capacity, but because systems were not designed to meet them where they are. They called for improved public understanding and targeting messaging to close the gap.

*“There’s a 20-year gap in mortality just within the city. People*,* over [place name] make their advanced care plan*,* and they put their powers of attorney in place*,* and they know about it. So*,* it’s educating the wider population who don’t get a chance to hear about it.” Participant 14*,* GP Clinical Advisor for Palliative and End-of-Life Care*.

Collectively, participants highlighted that current ACP frameworks were not sufficiently sensitive or adaptable to address diverse needs, raising concerns that they may inadvertently reproduce existing inequalities rather than reduce them.

#### Commissioning and resource constraints

ACP was often described as difficult to prioritise amid competing service demands. Workforce pressures and limited time were perceived as key barriers.

*“The job’s quite busy. I don’t get a lot of opportunity […] you’ve got two visits already and have to fit someone in because […] that’s the right care to do.” Participant 3*,* GP Lead for Palliative and End-of-Life Care*.

Several participants suggested ACP should be explicitly supported within commissioning and service specifications, ensuring time and resources are allocated to enable proactive discussions, however they highlighted that this did not happen.

*“We’ve moved from two separate contracts into one […] but pathways aren’t streamlined. They’ve all developed in ‘weird and wonderful ways.’ We need to look at them and try and really streamline those pathways.” Participant 8*,* Commissioner for Palliative and End-of-Life Care*.

Participants also emphasised that ACP needed to be realistic and proportionate to services available.

*“It’s all very well asking people what we want or where they want to die or the things that they do and don’t want to happen to them but*,* if we don’t have the services and the infrastructure to provide that care then it’s a bit like*,* you know*,* it’s like asking for your Christmas list knowing that it’s not going to happen.” Participant 12*,* Hospital Palliative Care Consultant*.

#### Fragmented care and unclear ownership

Participants highlighted fragmented care across multiple specialties and services. Clinicians often focused on single disease areas, leaving no one to “hold the whole picture”, contributing to delayed planning and conflicting advice.

*“The cardiologist wants to increase furosemide; the nephrologist stops it. Each is doing their best*,* but no one grabs the whole picture…. Each of these specialities is doing the best they can for this patient*,* but no one is grabbing the whole of it and going*,* ‘What needs to happen for this patient is…’ So they’re hiding in their specialities.” Participant 9*,* GP*,* Out-of-Hours Service*.

Participants also highlighted avoidance on both clinician and patient/family sides, further delaying conversations.

*“I think that it’s down to health professionals to… just not be afraid to have conversations that are potentially quite challenging*,* and could be quite triggering*,* and could evoke some*,* sometimes*,* unwanted responses from patients if they’re not in a place where they can hear it*,* because it can be really difficult*,* being on that side of it*,* as well.” Participant 18*,* Matron*,* Acute Response Team*.

#### Continuity and trust

Despite service fragmentation, participants emphasised continuity of relationships as essential for effective ACP. Trust enabled patients and families to engage with sensitive discussions. Where continuity was absent, ACP was more difficult, often delayed or avoided.

*“They want to see the same doctor. They don’t want to repeat the story twice. But our system*,* for various reasons*,* isn’t easy to fill that need.” Participant 3*,* GP Lead for Palliative and End-of-Life Care*.

Participants described the value of continuity in enabling holistic assessment and relationship-based planning.

*“Primary care was less pressured. The GP had really detailed and good relationships with patients. In terms of doing that holistic assessment*,* there was continuity.” Participant 7*,* Hospice Clinical Lead*.

However, continuity alone was not seen as sufficient if there was no shared plan or active coordination.

*“A nurse popping in once a week […] if they haven’t addressed the illness and come up with a plan*,* continuity of care is not helpful.” Participant 1*,* Community Palliative Medicine Consultant*.

#### Information sharing and IT interoperability

ACP documentation often failed to cross organisational boundaries, leading to duplication and crisis decisions. Out-of-hours and ambulance services were frequently unable to access ACP records.

*“It was all well and good*,* having a beautiful care plan*,* well documented on (named electronic healthcare system) or wherever*,* but the Ambulance Service don’t use (named electronic healthcare system).” Participant 19*,* Paramedic/ACP Coordinator*.

Participants also described the distress and inefficiency created when ACP information could not be accessed or transferred across settings.

*“We should be more joined up. The patient shouldn’t have to tell the journey or the story more than once or twice… We’ve got really good technologies nowadays to be able to share a support plan. But we’ve not got that technology yet.” Participant 17*,* Head of All Age Continuing Care*.

In the UK, processes such as ReSPECT (Recommended Summary Plan for Emergency Care and Treatment) have been developed to facilitate shared decisions between patients and clinicians in relation to emergency treatments, including cardiopulmonary resuscitation [36]. Participants noted the impact of a lack of transfer documentation from secondary care on ACP conversations:

*“At the moment*,* we don’t get an electronic ReSPECT form…there’s no documentation of any conversations that happen in hospital for us*,* and I think it’s distressing to keep repeating advanced care planning conversations.” Participant 14*,* GP Clinical Advisor for Palliative and End-of-Life*.

Participants stressed the need for interoperable systems that would make ACP documents visible across settings.

#### Training and professional readiness

Many professionals felt underprepared for ACP in MLTC, particularly in socially complex situations (e.g., poverty, unstable housing, language barriers, limited health literacy, family conflict, safeguarding concerns, or limited caregiver capacity) or where there were no clear prognostic milestones. A mismatch between clinicians’ confidence and ability was also highlighted:

*“They [clinicians] think they’re not doing it well. You often hear GPs going ‘Oh*,* I can’t have that conversation. I can’t do that’. Well*,* actually*,* the skills you need to have – either advanced care planning or palliative care – are actually the bread-and-butter skills of general practice. You just need to translate that. But it’s something scary*,* isn’t it?” Participant 3*,* Integrated Care Board Commissioner*.

Participants advocated for training in communication skills, cultural competence and ACP facilitation across the workforce.

### Theme 3: Delivering ACP in practice: frameworks, timing and communication

#### The role and limitations of ACP frameworks

Structured tools (such as emergency healthcare and treatment escalation plans) helped ensure preferences were visible during crises. However, when used rigidly or treated as an endpoint, participants felt ACP could become reduced to form completion (for example, DNACPR documentation) and overly focused on treatment escalation decisions. This reinforced the need to pace, revisit and broaden ACP so it remains relevant to the everyday lives of people living with MLTC.

*“ReSPECT is the very end of advanced care planning conversations. It’s*,* like*,* the really medical bit about whether you want to go into hospital or whether you want to be resuscitated. There’s a whole load of other information about what people’s wishes are about*,* where they’d like to be cared for*,* or even what happens to their dog if they’re unwell*,* what they’d like to eat*,* those sorts of things. Actually*,* those sorts of issues are often more important to patients than necessarily the DNACPR decision.” Participant 14*,* GP Clinical Advisor for Palliative and End-of-Life*.

Frameworks were described as most effective when used flexibly as prompts for dialogue, with personalised content reflecting everyday priorities as well as clinical decisions.

Participants also described instances where documentation was incomplete and overly focused on a single decision, limiting usefulness for person-centred planning.

*“[For hospital admission] sometimes it’s a ticked yes/no… just a name*,* NHS number*,* next of kin… but everything else*,* no*,* not filled in. It’ll definitely be signed to say ‘Do Not Resuscitate*,*’ but that’s also not helpful.” Participant 16*,* Advanced Paramedic*,* Palliative Care Champion*.

#### Triggers, timing and the importance of early ACP

Participants agreed ACP should be introduced early and revisited iteratively. In MLTC, this meant using practical triggers such as functional decline, recurrent admissions, carer strain or escalating treatment burden, rather than waiting for a single prognostic milestone.

*“It’s about having that conversation with the person and knowing and updating regularly what their issues are*,* because they change so rapidly as well.” Participant 22*,* Community Palliative Care Nurse*.

Embedding ACP within routine touchpoints (e.g., annual reviews, frailty assessments, post-discharge follow-ups) was recommended, particularly for patients who did not fit conventional palliative care referral pathways.

*“Long-term conditions are really important. I don’t think it’s given the emphasis and the support to navigate the nuances of palliation quite like other disease processes.” Participant 18*,* Matron*,* Acute Response Team*.

Early ACP was described as especially important in MLTC, where crises often arose suddenly and unpredictably.

*“It’s really important to try to get emergency healthcare plans in because I think that makes a big deal […] they [families] feel a little bit less stressed about it*,* a little bit more confident about keeping their relatives at home.” Participant 26*,* Community Matron*.

Participants also described the importance of ACP being “held” by someone who knew the person well, with suggestions that different roles might take this function depending on context.

*“Having someone that knows them I think is really important […] and maybe that’s*,* you know*,* almost like a key worker. So I think your key worker is going to be the GP or it might be the social worker*,* it might be mental health nurses*,* or dementia [nurses].” Participant 5*,* Community Geriatrician*.

Social prescribing and community nursing roles were also described as well-placed to support relational, contextual planning.

*“District nurses are in people’s homes […] they understand the context […] they’re also that link […] between the acute and community*,* and they often have quite good links with the primary care teams.” Participant 11*,* Public Health End-of-Life Community Engagement Lead*.

#### Compassionate communication and family inclusion

ACP was understood as a therapeutic communication process built on honesty, compassion and inclusion of families. Professionals described acknowledging uncertainty, exploring trade-offs and framing plans around “bad days” and practical contingencies. Trusting relationships were seen as central to meaningful ACP.

*“… each practice has an allocated specialist nurse. They tend to see their practice patients*,* So that’s really*,* really*,* important as well in that there’s it comes down to communication and relationships so that they and… they know the district nursing team’. Participant 3*,* Integrated Care Board Commissioner*.

Some participants described the need for clear, compassionate communication with families in acute deterioration, including naming dying explicitly.

*“…you have to sit down and really say*,* ‘Your mum’s actively dying*,* I am so sorry*,* but this is what the process is.’” Participant 16*,* Advanced Paramedic*,* Palliative Care Champion*.

Participants also linked ACP and preparation to wider experiences of dying and bereavement.

*“The importance of preparing… if you’re better prepared*,* you have a much better experience generally of dying. And if you have better preparation and a better experience of dying*,* you tend to have a better experience of grieving.” Participant 11*,* Public Health End-of-Life Community Engagement Lead*.

### Summary of Findings

ACP for advanced MLTC was described as important but frequently delayed or narrowed to crisis documentation. Participants attributed this to cumulative, hard-to-recognise decline, fragmented single-condition pathways and social circumstances that shape engagement and feasibility. They described effective ACP as earlier, iterative and embedded in routine care, focused on everyday priorities and caregiving capacity, and supported by continuity, clear responsibility and cross-service visibility of ACP records.

## Discussion

This study explored health and social care professionals’ perspectives on advance care planning (ACP) for people living with advanced multiple long-term conditions (MLTC). Across four English case-study regions, participants described ACP as necessary but difficult to deliver reliably within a system largely organised around single diseases and episodic care. Three themes were identified: Understanding the person living with MLTC and the importance of ACP, Systemic and organisational challenges to effective ACP, Delivering ACP in practice: frameworks, timing and communication.

### Why ACP in MLTC is distinct

A central finding was that ACP is experienced differently by both people with MLTC and the professionals providing the ACP, because deterioration is distributed across interacting conditions, often without a single dominant disease trajectory. This aligns with policy and conceptual work emphasising that MLTC is not simply the sum of conditions, but a pattern of interacting burdens (treatment, functional, cognitive, social) that creates distinctive planning challenges [3, 4]. Participants’ emphasis on “the bit in between that we miss” suggests that the challenge is not only prognostic uncertainty, but the absence of shared clinical cues for recognising cumulative decline.

Importantly, uncertainty should not be treated as a rationale to defer ACP. International consensus conceptualises ACP as an iterative process that can begin at any age or stage of illness and becomes more specific as health changes [10, 37]. Consistent with this, participants in this study argued for a shift from prognosis-based initiation to trigger-initiated ACP, starting and revisiting planning when observable markers accumulate (for example functional decline, repeated crises, escalating support needs, carer strain).

### “Treatment ceilings” and why the paperwork is not the ACP

Participants described a recurrent risk that ACP becomes narrowed to documen ting decisions about treatment escalation, rather than supporting ongoing conversations about what matters to the person. These findings also resonate with literature that questions whether ACP, in practice, can become overly focused on documentation and system-level requirements [17, 38]. Participants’ accounts of ACP being reduced to DNACPR decisions or form completion highlight a risk of planning processes prioritising clinical decision-making over patients’ social, relational and everyday concerns. This tension reflects an ongoing debate within the field as to whether ACP primarily serves patient-centred goals or organisational needs [39], with Unroe et al., 2016 [40] noting how honouring patient preferences for what matters to them *‘fulfills the promise of the advance care planning paradigm’* (p. 453).

UK developments such as ReSPECT were introduced partly because resuscitation decisions had become detached from broader goals-of-care discussions; ReSPECT aims to summarise personalised recommendations for emergency situations [36, 41]. In practice, these “treatment ceilings” are agreed limits on clinical escalation (for example admission to hospital or intensive care, ventilation, or cardiopulmonary resuscitation) and are often recorded in emergency care and treatment escalation plans. Our findings suggest that structured tools can improve visibility at points of crisis, but only when they are used to capture the output of wider, values-based dialogue rather than treated as the ACP itself.

Participants emphasised that, for people living with MLTC, ACP must extend beyond escalation decisions to address feasibility and everyday priorities, including safety, continuity, treatment and medication burden, carer sustainability, and place of care. This is consistent with evidence that ACP works best when implemented as a communication-and-review process over time, not simply as form completion, and is associated with better concordance between care and preferences and fewer unwanted high-intensity interventions, although effects vary by context [37, 42]. At the same time however, study participants discussed processes such as DNACPR and ReSPECT within interviews. This might suggest a level of dissonance between the system-level priorities of professionals and the person-centred approach: what matters to the patients themselves.

### Fragmentation as a product of health-system development

Participants repeatedly linked poor ACP delivery to fragmented pathways and diffuse responsibility. This is likely a structural consequence of how health and social care systems evolved: people typically enter through generalist care, but are then referred into specialist services and guideline-driven pathways organised around single conditions, with limited mechanisms for re-integration when needs become multi-dimensional. National guidance on multimorbidity explicitly highlights treatment burden, polypharmacy, multiple appointments and uncoordinated care as predictable consequences of single-disease approaches [43]. The Academy of Medical Sciences similarly identifies MLTC as a system-level challenge requiring reorientation from single-disease models toward person-centred, coordinated care [3]. Aligned with literature highlighting the importance of continuity in palliative care [44], our findings suggest that person-centred care is negatively impacted when ACP becomes “owned by nobody”; when no role or setting is resourced and authorised to hold the whole-person narrative across those fragmented pathways.

Rather than locating the solution solely in individual clinician skills, participants’ accounts imply the need for an identified consistent coordinating role that also acts as an ACP anchor (often in primary/community care) supported by interoperable information-sharing, so that preferences and priorities travel across services and out-of-hours contexts.

### Equity, inclusion and the risk of reproducing disadvantage

Professionals described inequities in access to ACP, emphasising that barriers were frequently systemic and professional, rather than reflecting patient reluctance. Evidence is consistent that documented ACP is less likely to be present for some minoritised ethnic groups, with multiple interacting causes including communication barriers and differential access to services [14]. Recent qualitative work in cancer care similarly describes practical and cultural barriers affecting ACP implementation for people from ethnic minority backgrounds [45]. Participants in this study highlighted mistrust, limited cultural confidence, and inadequate tailoring of ACP processes, implying that equity requires more than offering the same paperwork to everyone. A practical implication is that trigger-initiated ACP must be paired with equity-oriented delivery (for example, interpreting support, culturally safe communication, and proactive outreach through trusted routes).

### Relationship-based, trigger-initiated ACP as a pragmatic model

A coherent “best practice” model emerged from participants’ accounts: ACP works when it is relationship-based, started earlier, revisited iteratively, and made visible across settings. The distinctive contribution of this paper is the practical articulation of *how* to operationalise “earlier” in MLTC; through triggers grounded in function and support needs, not disease milestones. This is consistent with the broader demographic reality that palliative care need is rising and increasingly driven by chronic illness and complex trajectories, requiring integration across health and social care rather than reliance on specialist services alone [7, 9]. Integrated care for people with MLTC can be understood as a key element of person-centred care, as sustained therapeutic relationships support the accumulation of knowledge about the patient’s values, preferences and lived experience [46]. In turn this facilitates shared understanding, trust, and more individualised decision-making over time.

### Public understanding and normalisation

**Fig. 2:**
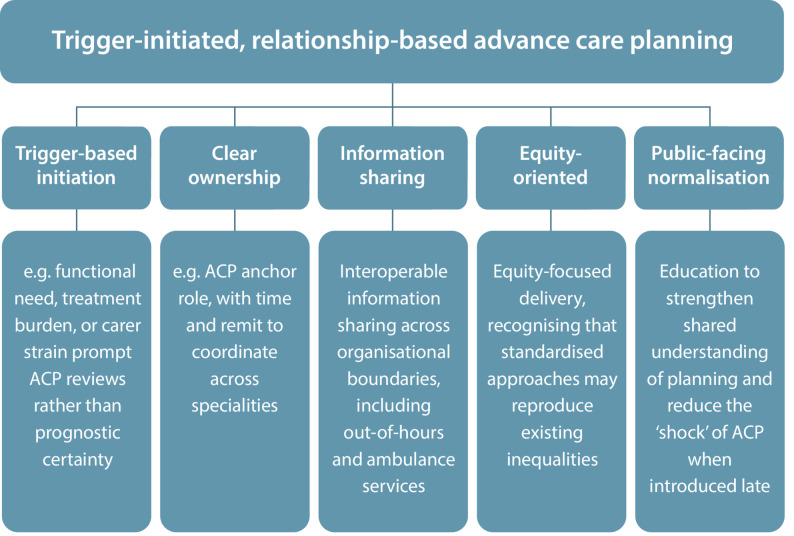
Implications for practice and policy

Participants also pointed to the limits of clinical interventions alone, arguing that ACP will remain fragile unless conversations about deterioration, dying and planning are more socially normalised. Public health palliative care approaches emphasise community capability and “death literacy” as complements to formal services [47, 48]. This supports the view that improving ACP for MLTC includes not only clinical triggers and documentation systems, but also broader efforts to build familiarity and confidence with planning long before crises occur. The practice and policy implications of the study findings are summarised in Fig. 2.

### Strengths and limitations

This study drew on a diverse range of professional roles across four English regions, and included the perspectives of out-of-hours staff and paramedics that directly illuminate why ACP visibility fails at the point of crisis. A limitation is that findings reflect professional accounts and may under-represent patient, family and community perspectives on what “good ACP” looks like in MLTC. In addition, while the sample included health and some social care-related roles, specialist social care practitioner perspectives were limited; future work should examine how social care settings and assessments could serve as key trigger points for ACP in MLTC. Finally, although a multiple case-study design enabled attention to context, accounts were broadly consistent and we did not identify stable contrasts between sites; this should be interpreted cautiously, as differences may exist but were not salient within these data.

## Conclusion

Advance care planning for people living with advanced multiple long-term conditions is both essential and structurally vulnerable. The challenge is not only clinical uncertainty, but a system organised around single-disease pathways that struggles to re-integrate care as multi-dimensional needs accumulate. A trigger-initiated model anchored in functional change and escalating support needs, delivered through trusted relationships, made visible across services and normalised in society offers a pragmatic route to earlier, more meaningful advance care planning for this growing population.

## Supplementary Information

Supplementary Material 1.

Supplementary Material 2.

## Data Availability

The data analysed in this study are not publicly available due to confidentiality agreements and the sensitive nature of the interview content. Anonymised extracts are presented within the manuscript. Requests for access to anonymised data may be considered on reasonable request and should be directed to the corresponding author (mark.adley@newcastle.ac.uk), subject to appropriate governance approvals.

## Abbreviations

ACP: Advance Care Planning
AHP: Allied health professional
CEO: Chief Executive Officer
DNACPR: Do Not Attempt Cardiopulmonary Resuscitation
GP: General Practitioner
MLTC: Multiple Long-Term Conditions
NHS: National Health Service
ReSPECT: Recommended Summary Plan for Emergency Care and Treatment
