# Supplementary material for: Advance care planning for patients with advanced multiple long-term conditions: a qualitative thematic analysis of health and social care professionals’ perspectives
Source: BMC Palliat Care. 2026 Jun 4;25:236. doi: 10.1186/s12904-026-02173-2 (PMC13455371; doi:10.1186/s12904-026-02173-2)
Supplement: Supplementary file 1 [file 12904_2026_2173_MOESM1_ESM.pdf]

Supplementary Material 2

Interview Topic Guide

Role/ Service details

Can you start by telling me a bit about the role you hold within your service and the service that you work in?

Can you describe the patient population that you look after and the reach of your service (local/regional)?

Introductory prompts for interview

1. When we contacted you to discuss the palliative care needs of patients with multiple long-term conditions, what were your first thoughts? (or if they reached out) What was it that interested you about this research?

(Note: we are defining MLTC this as 2 or more chronic conditions)

2. What aspects of care and support do you think this group would benefit from in the last year of their life? Are there changes to this as death approaches?

Or

In your view, what are the palliative care needs of people living with MLTC in the last year of life? Do these change as death approaches?

3. Thinking of a typical patient with multiple LTCs (eg lung disease, heart failure and kidney failure) - how do you see their journey through your service?
4. Have you encountered any challenges in providing good care for this group?
5. Do you have any examples of where challenges or barriers have been overcome?

Service innovation/design

6. What do you see as the 'ideal' service for this group?
7. Can you expand on why you have identified these key aspects?
8. Who do you see as the key players/collaborators in providing care
9. Where do you think care should be provided?
10. What role do families/carers play in care? How can we support this?

If not already mentioned:

11. What do you think about the role of advance care planning?

Suggested topics to cover

Palliative care needs

1. What palliative care needs do people with MLTCs have?
2. How do these needs evolve as the end-of-life approaches?

Approaches to providing care – what is the ideal, and what are your experiences of current care

1. Considering your experiences – what do you think are the key components of a service that provides palliative care to patients with MLTC?
2. Can you expand on why you have identified these components?
3. What might the ideal 'patient journey' look like in such a service? (if describing a service where you already work – tell us what is working well and what is working less well)
4. Considering your experiences – what are the barriers to providing palliative care to this group?
5. How do you/could you involve family/caregivers in this care provision?

The Workforce

1. WHO should provide this care, and what training/qualifications should they have?
2. WHERE should this care be provided?
3. WHEN should this care be provided - what should the triggers for involvement of this workforce be?
4. Who should be collaborating with this workforce to provide care, and have you seen any examples of effective collaboration between services to improve the experience of this group?

Care Coordination

1. How can we make services more integrated for people with MLTC at the end of life?
2. If you currently work within a service – do you have single point of access?
3. How do you facilitate continuity of care (e.g. when patients are admitted), and what are the barriers to continuity of care?

Effectiveness

1. How can we show that a service/intervention is effective?
2. Do you measure outcomes in the service in which you currently work/your current role?

Optional additional areas for reflection:

- ACP: How are goals of care conversations performed for people with MLTC?
- How do you use digital technology in caring for those with MLTC?
- How can we identify patients appropriate for a palliative care approach?
