# Supplementary material for: Advance care planning for patients with advanced multiple long-term conditions: a qualitative thematic analysis of health and social care professionals’ perspectives
Source: BMC Palliat Care. 2026 Jun 4;25:236. doi: 10.1186/s12904-026-02173-2 (PMC13455371; doi:10.1186/s12904-026-02173-2)
Supplement: Supplementary file 2 [file 12904_2026_2173_MOESM2_ESM.pdf]

Supplementary Material 1

Consolidated criteria for reporting qualitative research (COREQ)

No	Item	Guide questions/description	Reported on page no.
**Domain 1: Research team and reflexivity**			
Personal Characteristics			
1	Interviewer/facilitator	Which author/s conducted the interview or focus group?	7
2	Credentials	What were the researcher's credentials? *E.g. PhD, MD*	7
3	Occupation	What was their occupation at the time of the study?	7
4	Gender	Was the researcher male or female?	7
5	Experience and training	What experience or training did the researcher have?	7
Relationship with participants			
6	Relationship established	Was a relationship established prior to study commencement?	7
7	Participant knowledge of the interviewer	What did the participants know about the researcher? e.g. *personal goals, reasons for doing the research*	7
8	Interviewer characteristics	What characteristics were reported about the interviewer/facilitator? e.g. *Bias, assumptions, reasons and interests in the research topic*	7
**Domain 2: study design**			
Theoretical framework			
9	Methodological orientation and Theory	What methodological orientation was stated to underpin the study? *e.g. grounded theory, discourse analysis, ethnography, phenomenology, content analysis*	6
Participant selection			
10	Sampling	How were participants selected? *e.g. purposive, convenience, consecutive, snowball*	7
11	Method of approach	How were participants approached? e.g. *face-to-face, telephone, mail, email*	7
12	Sample size	How many participants were in the study?	7
13	Non-participation	How many people refused to participate or dropped out? Reasons?	7
14	Setting of data collection	Where was the data collected? e.g. *home, clinic, workplace*	8
15	Presence of non-participants	Was anyone else present besides the participants and researchers?	8
16	Description of sample	What are the important characteristics of the sample? *e.g. demographic data, date*	8, 10

Data collection			
17	Interview guide	Were questions, prompts, guides provided by the authors? Was it pilot tested?	7
18	Repeat interviews	Were repeat interviews carried out? If yes, how many?	8
19	Audio/visual recording	Did the research use audio or visual recording to collect the data?	8
20	Field notes	Were field notes made during and/or after the interview or focus group?	8
21	Duration	What was the duration of the interviews or focus group?	7
22	Data saturation	Was data saturation discussed?	8
23	Transcripts returned	Were transcripts returned to participants for comment and/or correction?	8
**Domain 3: analysis and findings**			
Data analysis			
24	Number of data coders	How many data coders coded the data?	8
25	Description of the coding tree	Did authors provide a description of the coding tree?	n/a
26	Derivation of themes	Were themes identified in advance or derived from the data?	9
27	Software	What software, if applicable, was used to manage the data?	8
28	Participant checking	Did participants provide feedback on the findings?	9
Reporting			
29	Quotations presented	Were participant quotations presented to illustrate the themes / findings? Was each quotation identified? e.*g. participant number*	9
30	Data and findings consistent	Was there consistency between the data presented and the findings?	9
31	Clarity of major themes	Were major themes clearly presented in the findings?	9
32	Clarity of minor themes	Is there a description of diverse cases or discussion of minor themes?	9
